# Diode laser assisted horizontal bone defect regeneration in the treatment of peri‐implantitis

**DOI:** 10.1002/ccr3.3741

**Published:** 2021-01-03

**Authors:** Ghassan Habash, Soher Nagi Jayash

**Affiliations:** ^1^ Periodontology Department Faculty of Dentistry AlQuds University Jerusalem Palestine; ^2^ University of Health Science Lahor Pakistan; ^3^ School of Dentistry University of Birmingham Birmingham UK

**Keywords:** bone regeneration, diode laser, horizontal bone loss, peri‐implantitis

## Abstract

Our findings show the use of a diode laser to remove inflamed tissue and regenerate the horizontal bone defects around the implant. These findings could be applied in the clinic.

## Graphical abstract

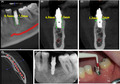

## 1 INTRODUCTION

This paper describes a clinical case of peri‐implantitis treatment using a diode laser. In the present report, a peri‐implantitis case with bone resorption up to two‐thirds of the implant healed by laser and nonsurgical treatment. It is recommended to use a diode laser for horizontal bone regeneration.

Most titanium implants were constructed with a rough surface to rise the implant‐bone contact areas and fix the implant in the alveolar bone. However, this surface impedes the complete elimination of bacteria from implant surfaces and may lead to peri‐implantitis. Peri‐implantitis is an inflammatory process that affects the tissues around the implant causing bleeding, suppuration, increased probing depth, and radiographic bone loss. It was reported that the inflammation is more prominent and the inflammatory process is more aggressive around the dental implant than around the natural tooth.^1^ Peri‐implant defects can be classified as class I ‐ demonstrates moderate horizontal bone loss and minimal intra‐bony component. This represents an early stage of peri‐implant breakdown, class II ‐ presents moderate to severe horizontal bone loss and minimal intrabony component, class III ‐ presents minimal to moderate horizontal bone loss with an advanced circumferential intra‐bony lesion, in class IV‐ more complicated defects with moderate horizontal bone loss and more an advanced circumferential intra‐bony lesion, furthermore, buccal and/or lingual plate has been lost.^2^ Therapeutic goals in the treatment of peri‐implantitis are removal of inflammation, improved bony attachment, and regeneration. To achieve these goals; any foreign cells and toxins should be removed from the implant surface. Subsequently, the tissue inflammation is reduced, and the healthy tissue may attach and contact the implant surface again. Thus, decontamination and detoxification of the implant surface are important.^3^ Some treatments have been produced for cleaning and decontamination of the implant surfaces via metal or plastic curettes or ultrasonic scalers that may cause damage to the surface of implants so it was not recommended.^4^ Antibacterial agents and local or systemic antibiotics can be helpful in the peri‐implantitis treatment.^5^ Most of these treatments are predominantly centralized around the elimination of bacterial infection. The regenerative approach for peri‐implantitis treatment is a rising problem. The ideal treatment for peri‐implantitis is the regeneration of soft and hard tissues supporting dental implants.^6^ Laser technology is developing with broad characteristics that are available for use in tissue healing, diode laser is used in different medical applications especially in photodynamic therapy and noninvasive surgical procedures. The diode laser is usually used more than other lasers because of its reliability, brightness, and compact size. Also, low level laser therapy (LLLT) can be used to enhance wound healing and effect in modulating locally and systemically. LLLT is sometimes known as photobiomodulation and its effect causing by photochemical not thermal, and the light triggers biochemical changes within cells. The principle behind the application of low level lasers is light energy application with biomodulatory capacity on body cells leading to increase cell activity.^7^ Laser treatments were used in different application for bone treatment. The assessment of the effects of laser therapy on horizontal bone regeneration is an important field of investigation. This case report aims to describe/assess and added clinical benefits of laser in the treatment of peri‐implantitis.

## 2 CASE REPORT

### 2.1 Methods

**FIGURE 1:**
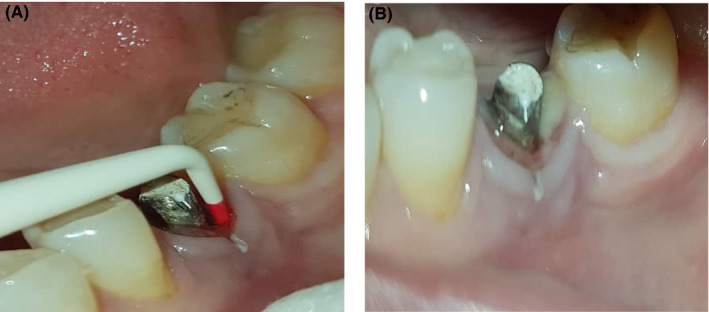
A, Pocket depth around the implant. B, Pus discharge around implant

**FIGURE 2:**
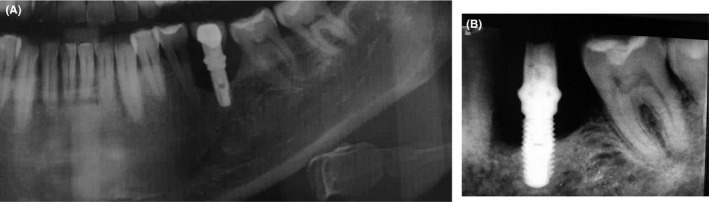
A, panoramic radiograph of implant site B, Intraoral periapical radiograph of implant site

**FIGURE 3:**
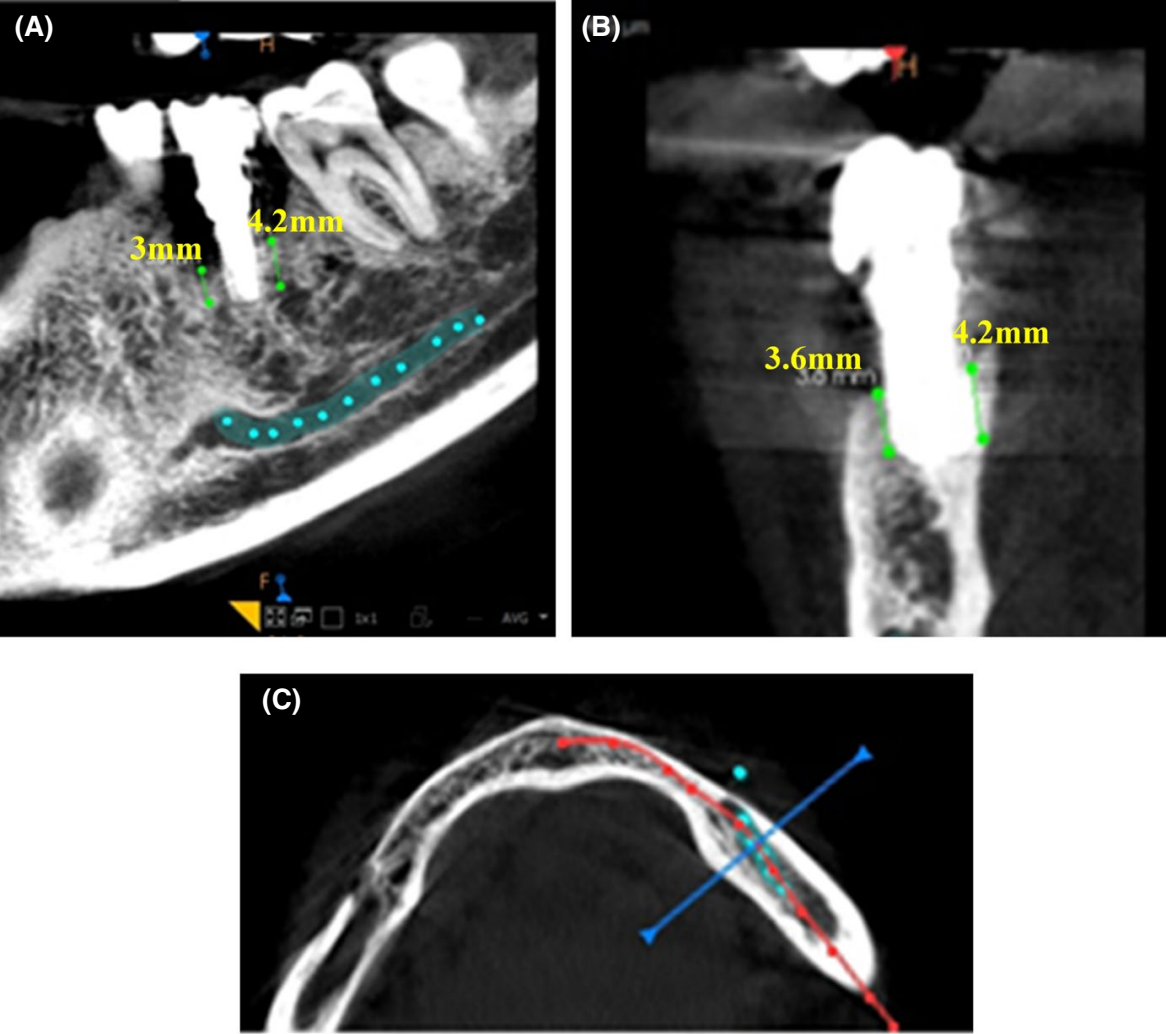
The CBCT imaging of a peri‐implantitis case showing bone resorption in mesiodistal view (A), buccolingual view (B) and axial view (C) around dental implant

54‐year‐old female patient, with no significant past medical history and daily takes vitamin D (5000 I.U), presented to the dental office with chief complaint pus in the implant site with a history of implant placement for lower left mandibular first molar 15 years back. On examination, peri‐implant mucosa was inflamed, tender on palpation pus discharge around implant ill‐fitting crown (Figure 1). Panoramic and intraoral periapical radiographs showed crestal and horizontal bone loss around the implant (Figure 2). Also, cone beam computed tomography (CBCT) revealed bone loss around the implant suggestive of class II peri‐implant defect. The bone resorption was up to two‐thirds of the implant. The alveolar bone‐height measurements from CBCT images bone were 3, 4.2, 3.6, 4.2 mm in mesial, distal, lingual, and buccal sides of implant respectively (Figure 3).

Peri‐implant regenerative therapy was planned following patient consent for the treatment. The treatment started by removal of the ill‐fitting crown. The pocket depth was 8 mm, nonsurgical debridement was done after achieving adequate local anesthesia by using manual titanium and plastic curettes, and excess cement removed. The air powder polishing (Prophy powder, US) was done followed by laser decontamination using hot tip (1 wt) with pulsating mode keep moving in and out for 30 seconds. Peri‐implant bone was irradiated with low level laser therapy using 810 nm diode laser (QuickLase), in contact pulsating wave mode (0.1 W) in an apico‐coronal, back and forth directions for 45 seconds then manual curettage and debridement followed by 12% chlorhexidine lavage. Postoperative instructions were given and Augmentin 500 mg and 250 mg flagyl for 1 week and Chlorhexidine mouthwash for 14 days twice daily. The patient was reviewed after 45 days. No fresh complaints were observed in relation to the treated site.

## 3 RESULTS

**FIGURE 4:**
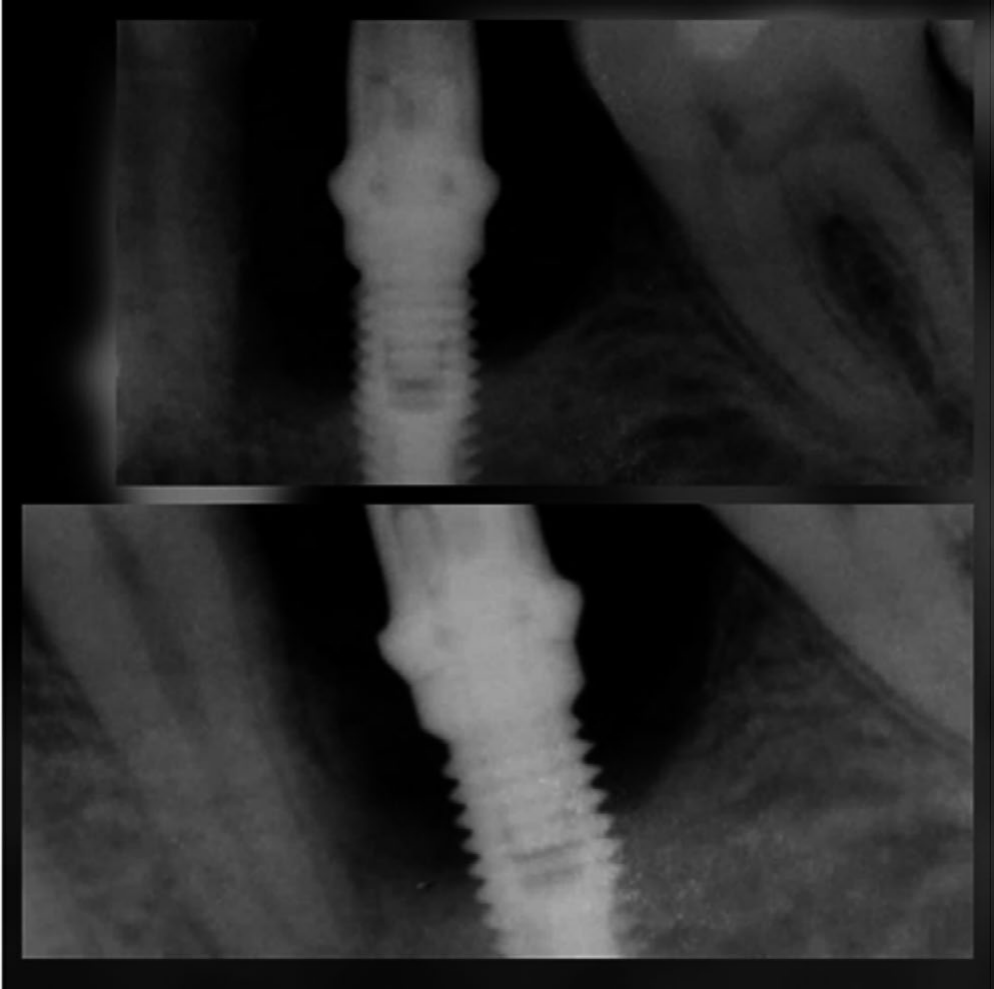
Intraoral periapical radiograph of implant site 2 mo after treatment

**FIGURE 5:**
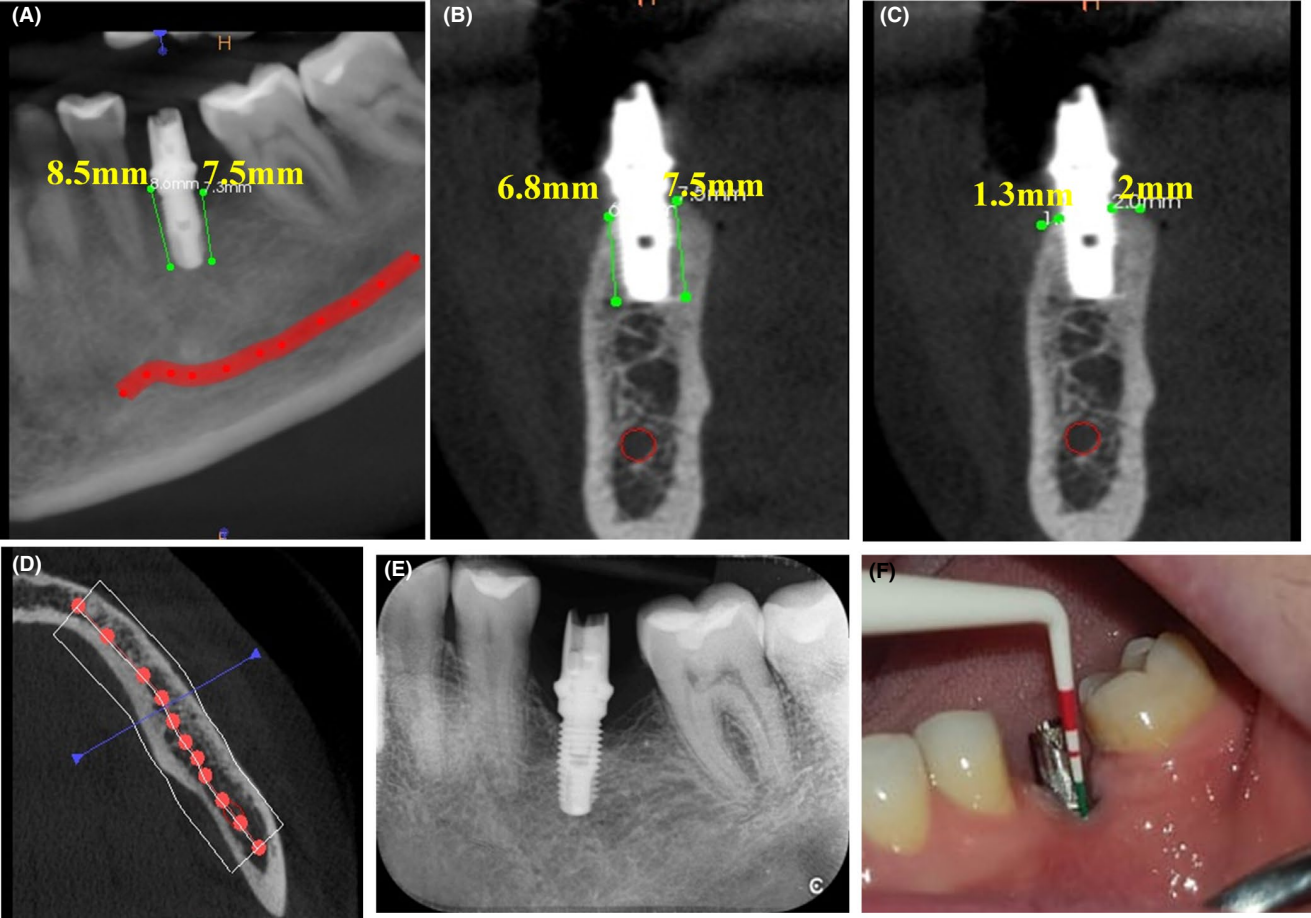
The CBCT imaging of a peri‐implantitis case showing bone healing in mesiodistal view (A), buccolingual view (B,C) and axial view (D) around dental implant, Intraoral periapical radiograph of implant site (E), pocket depth around the implant (F) 1 y after treatment

On 2‐month evaluation, intraoral periapical radiograph revealed bone lesion healing (Figure 4). On 1‐year evaluation, CBCT and intraoral periapical radiograph revealed complete bone fill and the pocket depth reduced to 1 mm. The alveolar bone‐height measurements from CBCT images bone were 8.5, 7.5, 6.8, 7.5 mm in mesial, distal, lingual, and buccal sides of implant respectively. The bone thickness adjacent to dental implant was 1.3, 2 mm on lingual and buccal sides of implant respectively (Figure 5).

## 4 DISCUSSION

Replacement of missing teeth with dental implants has become a standard procedure in modern day dentistry. Many clinical studies indicate that after 9‐14 years post‐implant placement, peri‐implant lesions are a common clinical issue. Peri‐implantitis is a common cause of implant loss which has some resemblance to periodontitis. Although, there are some exceptions that require the use of a different treatment technique. Nowadays, the various laser wavelengths are commonly used in implantology. However, the utilizing the high‐power laser systems leading to increase in the temperature of treated tissues that may require to use laser with low level laser to provide a proper result on lesion without having a harmful effect on surgical site.^8^, ^9^ The benefit of using laser around the implant over other techniques is their ability to reach hard areas around the implant to achieve better sterilization and debridement. The diode laser has a fine diode tip that removes the granulation tissue and does site sterilization without the need to remove the additional bone for treatment success and does not cause any alterations on polished and coated surfaces of implant.^10^ Numerous in vitro and in vivo studies have been focused on the use of LLLT for oral and periodontal purposes. The current case report describes the clinical benefits of photobiomodulation in the treatment of peri‐implantitis. LLLT's effect on the viability of osteocyte and tissue regeneration in peri‐implant bone were evaluated using a 100‐mW diode laser with 690 nm wavelength. Their results revealed a significantly higher percentage of osteocytes around implant subjected to laser irradiation immediately after implant placement compared to control. This is confirmed a positive effect of LLLT on the osseointegration.^11^ In the same line, diode laser had a bio‐stimulating effect on osteoblasts in vitro and this is may be effect on osseointegration of dental implants.^12^ Also, animal studies showed that LLLT stimulates interaction between implant and bone, so resulting more mechanical strength on the first 8 weeks.^13^ Another study reported a positive effect of LLLT treatment on osseointegration of dental implants placement with sinus augmentation, and enhance tissue healing.^14^, ^15^ In Pai et al study,^16^ peri‐implant infrabony defects showed adequate bone fill 6 months postoperatively after laser decontamination and bone augmentation. In the present case report, peri‐implant infrabony defects showed adequate bone fill 1 year postoperatively after only laser therapy. Using the described protocol, the authors were able to decontaminate the implant surface efficiently and induce higher levels of cytokines eg, transforming growth factor beta‐1 which promotes bone and soft tissue and bone mineralization. On the other hand, the patient takes calcium/vitamin D daily that may enhance bone healing. However, the effects of vitamin D on bone healing are still not clear, a number of studies highlight the importance of vitamin D in bone healing. The vitamin D has an anabolic effect on osteoblasts and can to increase bone growth and bone density. Also, some studies revealed adequate calcium/vitamin D is important to provide sufficient calcium for mineralization to prevent bone loss.^17^, ^18^, ^19^, ^20^, ^21^, ^22^

In this case report, complete healing and bone regeneration for horizontal bone defect around the implant which is observed by increase 3‐4 mm bone height and 1‐2 mm in bone thickness after one year of treatment. This is approving the effectiveness of nonsurgical treatment and LLLT in the treatment of peri‐implantitis.

## 5 CONCLUSION

This case report showed that a diode laser (810 nm) can be used for the debridement of the soft tissues around the implants. Low Level Laser Therapy using diode laser has possible bio‐stimulating effects which might be used in the treatment of peri‐implantitis and re‐osseointegration of dental implants. Further studies on the application of this method will show the beneficial effects of laser therapy and vitamin D supplement in the treatment of peri‐implantitis.

## CONFLICT OF INTEREST

The authors declare that there are no competing interests.

## AUTHORS' CONTRIBUTIONS

GH: performed laser treatment and collect the data. SJ: wrote the manuscript and data analysis.

## ETHICAL APPROVAL

Patient consent to participate is signed before starting the treatment and submitted as supplementary1. However, ethical Approval is not applicable.

## CONSENT FOR PUBLICATION

Consent for publication is signed and submitted as supplementary1.

## Data Availability

All supporting data are available.
